# Attribute nonattendance in COVID‐19 vaccine choice: A discrete choice experiment based on Chinese public preference

**DOI:** 10.1111/hex.13439

**Published:** 2022-01-20

**Authors:** Jianhong Xiao, Fei Wang, Min Wang, Zegang Ma

**Affiliations:** ^1^ School of Tourism and Geography Science Qingdao University Qingdao Shandong China; ^2^ Business College Qingdao University Qingdao Shandong China; ^3^ School of Basic Medicine Qingdao University Qingdao Shandong China

**Keywords:** attribute nonattendance, Chinese public, COVID‐19 pandemic, discrete choice experiment, preference heterogeneity, vaccine, willingness to pay

## Abstract

**Objectives:**

The global coronavirus disease 2019 (COVID‐19) pandemic has not been well controlled, and vaccination could be an effective way to prevent this pandemic. By accommodating attribute nonattendance (ANA) in a discrete choice experiment (DCE), this paper aimed to examine Chinese public preferences and willingness to pay (WTP) for COVID‐19 vaccine attributes, especially the influence of ANA on the estimated results.

**Methods:**

A DCE was designed with four attributes: effectiveness, protection period, adverse reactions and price. A random parameter logit model with an error component (RPL‐EC) was used to analyse the heterogeneity of respondents' preferences for COVID‐19 vaccine attributes. Two equality constraint latent class (ECLC) models were used to consider the influence of ANA on the estimated results in which the ECLC‐homogeneity model considered only ANA and the ECLC‐heterogeneity model considered both ANA and preference heterogeneity.

**Results:**

Data from 1,576 samples were included in the analyses. Effectiveness had the highest relative importance, followed by adverse reactions and protection period, which were determined by the attributes and levels presented in this study. The ECLC‐heterogeneity model improved the goodness of fit of the model and obtained a lower probability of ANA. In the ECLC‐heterogeneity model, only a small number of respondents (29.09%) considered all attributes, and price was the most easily ignored attribute (64.23%). Compared with the RPL‐EC model, the ECLC‐homogeneity model obtained lower WTPs for COVID‐19 vaccine attributes, and the ECLC‐heterogeneity model obtained mixed WTP results. In the ECLC‐heterogeneity model, preference group 1 obtained higher WTPs, and preference groups 2 and 3 obtained lower WTPs.

**Conclusions:**

The RPL‐EC, ECLC‐homogeneity and ECLC‐heterogeneity models obtained inconsistent WTPs for COVID‐19 vaccine attributes. The study found that the results of the ECLC‐heterogeneity model considering both ANA and preference heterogeneity may be more plausible because ANA and low preference may be confused in the ECLC‐homogeneity model and the RPL‐EC model. The results showed that the probability of ANA was still high in the ECLC‐heterogeneity model, although it was lower than that in the ECLC‐homogeneity model. Therefore, in future research on DCE (such as the field of vaccines), ANA should be considered as an essential issue.

**Public Contribution:**

Chinese adults from 31 provinces in mainland China participated in the study. All participants completed the COVID‐19 vaccine choice questions generated through the DCE design.

## 1 INTRODUCTION

The coronavirus disease 2019 (COVID‐19) pandemic was caused by severe acute respiratory syndrome coronavirus 2 (SARS‐CoV‐2), a new virus that was not known before 2019. The pandemic has had a serious impact on the health system, and no individual was immune to the virus at the beginning of this pandemic, because of which the pandemic has still not been well controlled, with rising numbers of infections and deaths in many countries. There are two ways to control the pandemic: Multiple lockdowns that happened in the beginning or effective vaccination to achieve global herd immunity.

China has achieved great success in vaccine research and development since the outbreak of the COVID‐19 pandemic. ‘But bringing a vaccine to market is only half the challenge; also critical is ensuring a high enough vaccination rate to achieve herd immunity’.^1^ Although the public acceptance rate of the COVID‐19 vaccine in China is extremely high compared with that in other countries,^2^ studies show that the Chinese public acceptance rate of the vaccine has a downward trend.^3^ Wang et al.^3^ studied the changes in the Chinese public acceptance of the COVID‐19 vaccine at different stages of the pandemic, and the results revealed that 91.9% of people were willing to be vaccinated against COVID‐19, and 58.3% wanted to be vaccinated immediately in March 2020. However, by November–December 2020, the Chinese public acceptance of the vaccine and the proportion of people who wanted to be vaccinated immediately dropped to 88.6% and 23.0%, respectively. With the persistence of the pandemic, the continuous variation of SARS‐CoV‐2 and the acceleration of vaccine development,^4^, ^5^, ^6^ people developed major doubts about the effectiveness and safety of the COVID‐19 vaccine.^7^ Therefore, the achievement of a vaccination rate of 75%–90% to achieve herd immunity^8^ in China may be a considerable challenge.^9^ It is crucial to understand the factors influencing vaccination and trade‐offs among these factors, especially the preferences for COVID‐19 vaccine attributes. To better understand the preferences, we include price attribute in this study, which is used to understand the relative value of the nonprice attributes.

The discrete choice experiment (DCE) is a widely used method in nonmarket valuation; its popularity is increasing because it can evaluate multiple attributes of a product simultaneously.^10^ The theoretical basis for DCE comes from random utility theory and Lancaster's^11^ consumer theory. According to Lancaster's consumer theory, the utility of the COVID‐19 vaccine chosen by respondents does not come from the COVID‐19 vaccine itself but from the multiple attributes of the COVID‐19 vaccine, which are selected to be comprehensive and complete. DCE has been used to assess respondents' preferences and willingness to pay (WTP) for the attributes of vaccines such as the meningococcal vaccine,^12^ the Tdap vaccine^13^ and the human papillomavirus vaccine.^14^, ^15^ Researchers have focused on respondents' preferences or WTP for COVID‐19 vaccine attributes since the outbreak of the COVID‐19 pandemic.^7^, ^16^, ^17^, ^18^, ^19^

DCE generally assumes that respondents can process all the information provided in each choice set when making choices and then choose their most preferred alternative.^20^, ^21^, ^22^, ^23^ However, a growing number of studies have begun to focus on the attribute nonattendance (ANA) phenomenon, in which respondents do not consider all the information provided when making choices, resulting in ignoring of some attributes.^24^, ^25^ ANA has been widely considered in studies related to health,^26^, ^27^ transportation,^28^, ^29^, ^30^ environment,^31^, ^32^ food,^33^, ^34^ agriculture^23^, ^35^ and other fields. Most studies have shown that a model considering ANA would obtain better goodness of fit and higher or lower WTP.^35^, ^36^, ^37^, ^38^ Little to no attention has been paid to ANA in the field of vaccines. An exception is Iles et al.,^39^ who considered ANA in their study of WTP for the contagious bovine pleuropneumonia vaccine in Samburu County, Kenya. To our knowledge, studies on vaccines for human use, including the COVID‐19 vaccine, have not considered ANA. Respondents are more likely to adopt simplified heuristics when they are unfamiliar with the research object or the choice tasks are complex.^40^, ^41^ In these cases, the influence of ANA on the research results should be considered. The COVID‐19 vaccine studied in this paper was relatively unfamiliar to the respondents. Therefore, this case study is actually an application of ANA to the COVID‐19 vaccine.

There may be two main reasons for ignoring attributes. First, respondents ignore some attributes by adopting simplified heuristics to reduce the cognitive burden due to the complexity of choice tasks, which reflects the real ANA.^26^, ^28^, ^42^ Second, respondents ignore some attributes because these attributes are not important to them, or are of low importance, which reflects the preference heterogeneity of respondents.^26^, ^27^, ^43^ Traditional processing models for ANA, such as the equality constraint latent class (ECLC) model considering only ANA, would lead to confusion between ANA and preference heterogeneity and may incorrectly identify respondents with low preference as nonattenders.^24^, ^44^ Therefore, this paper adopts an ECLC model that takes both ANA and preference heterogeneity into account to obtain more reliable results.

The first objective of this paper was to determine the Chinese public preferences and WTP for COVID‐19 vaccine attributes using DCE. The second objective was to take the COVID‐19 vaccine as a case to study the impact of ANA on DCE estimation results. Specifically, we focus mainly on the following three points: Whether considering ANA will improve the goodness of fit of the model; whether considering preference heterogeneity will reduce the probability of ANA; and whether the model considering ANA and preference heterogeneity will yield inconsistent WTP results with the model without considering ANA and improve the reliability of the modelling results.

## 2 METHODS

### 2.1 Selection of the attributes and levels for DCE

The design of the DCE follows International Society for Pharmacoeconomics and Outcomes Research guidelines.^45^ First, the research team reviewed the related literature, including papers on respondents' preferences for the COVID‐19 vaccine and other vaccines using DCE, as well as literature on the influencing factors of COVID‐19 vaccination. Based on the information obtained from the literature review, the research team held several discussions, and the four most frequently mentioned attributes that impact vaccine preference were selected: effectiveness,^46^, ^47^, ^48^ protection period,^16^, ^17^, ^18^ adverse reactions^16^, ^49^ and price.^16^ The selection of attribute levels was based on existing studies on the COVID‐19 vaccine and official reports of the COVID‐19 vaccines announced in China. Specifically, the effectiveness of the Sinopharm COVID‐19 vaccine reached 79.34% when it conditionally entered the market.^50^ Clinical trials of the Sinovac vaccine, CoronaVac, in Turkey and Indonesia yielded 91.25% and 65.3% effectiveness, respectively.^51^ Therefore, the effectiveness levels of this study were 65%, 80% and 95%. The protection period is still uncertain due to the short development time of COVID‐19 vaccines. However, Sinopharm predicted that the protection period of its vaccines may reach 1–3 years.^52^ Based on this information, we selected protection periods of 1, 2 and 3 years. There were mild adverse reactions and no adverse reactions,^16^, ^53^, ^54^ and the mild adverse reactions manifested mainly as local pain, redness and swelling at the injection site, transient low‐grade fever, fever, and so forth.^55^ Then, we invited two medical experts to participate in a discussion on the selection of attributes and levels. After the discussion, we used the above attributes and levels to build choice sets, and the price attribute levels selected were based on the pilot survey. Otherwise, other related information of the vaccine was specified in the questionnaire; that is, the hypothetical scenario positioned the COVID‐19 vaccine as a Chinese vaccine that requires two injections with an interval of 14–28 days between them.^56^ The attributes and levels can be found in Appendix S1.

### 2.2 Survey design

**Figure 1:**
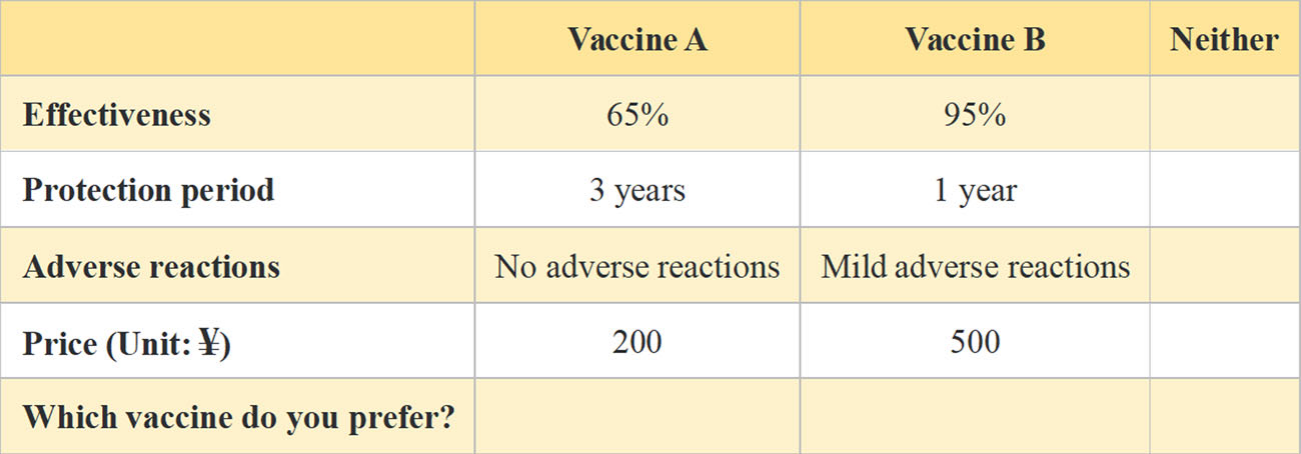
An example of a choice set

The full‐factor design produced 108 (3×3×2×6) hypothetical COVID‐19 vaccines, resulting in 5,778 (${∁}_{108}^{2}$) different choice sets. Taking the difficulty of sample collection for 5,778 hypothetical scenarios into account, this article used an orthogonal experimental design to produce eight choice sets. To simulate the real market and reduce respondents' protest bias, we added the option of neither choice in each choice set, which means an opt‐out option.^12^, ^19^ Additionally, the eight choice sets were randomly divided into two blocks in the questionnaire to mitigate the cognitive burden on respondents^57^; hence, each respondent faced four choice sets. An example of a choice set is shown in Figure 1.

The first section of the questionnaire was the DCE, which first introduced the hypothetical scenarios in detail and then asked respondents to make choices among four choice sets. The second section collected respondents' demographic characteristics (age, gender, marital status, number of children, city level and region), socioeconomic characteristics (education, average monthly income in 2019, and whether they were employed in a medical‐related industry) and health status (whether they had chronic diseases).

### 2.3 Data collection

A 2‐day pilot survey was conducted from 25 to 26 January 2021, to test respondents' understanding of attributes and levels of the COVID‐19 vaccine and to decide the price levels. The formal survey was conducted from 28 to 31 January 2021, and adopted the snowball sampling method (specific information on snowball sampling can be found in Appendix S2). All questionnaires were developed using Wenjuanxing (http://www.wjx.cn). The survey respondents included online users who were older than 18 years of age in 31 provinces in mainland China. The minimum sample size required for the DCE survey was calculated using the formula *N* = 500 × *L*/(*J* × *S*) recommended by Orme,^58^ where *N* is the sample size required for each version of the questionnaire, *L* is the largest number of attribute levels among all attributes (6), *J* is the number of alternatives in each choice set (2) and *S* is the number of choice sets in each version of the questionnaire (4). According to this calculation, at least 375 (500 × 6/[2 × 4]) questionnaires were required for each version. A total of 1,583 samples were collected in this survey, among which seven samples were excluded from the analyses since they did not come from any of the 31 provinces in mainland China. Data from 1,576 samples were included in the analyses, and a total of 6,304 observations were obtained.

### 2.4 Statistical analyses

In this paper, a random parameter logit model with an error component (RPL‐EC) was used to study the heterogeneity of the respondents' preferences for COVID‐19 vaccine attributes. The RPL‐EC model relaxes the limitation on the independence of irrelative alternatives and assumes that the respondents' preferences for the vaccine are heterogeneous by assuming a random distribution of coefficients.^59^, ^60^ This paper adopts the distribution form commonly used in previous studies; the nonprice attributes are assumed to be normally distributed, the price attribute is assumed to be constant and 1,000 Halton draws are used.^40^, ^61^, ^62^, ^63^

This paper specifies a common random error component in the random utility of the hypothetical options of vaccine 1 and vaccine 2 to capture any additional variance between the two hypothetical vaccine options.^33^, ^38^, ^64^ In the RPL‐EC model, the utility of respondent n choosing alternative COVID‐19 vaccine j from choice set t is

(1) $$U_{\mathrm{njt}}=\left\{\begin{array}{l} \beta_{n}^{\prime }X_{\mathrm{njt}}+\mu_{n}^{\prime }e_{\mathrm{nj}}+\varepsilon_{\mathrm{njt}},j=1,2\\ \mathrm{ASC}+\beta_{n}^{\prime }X_{\mathrm{njt}}+\varepsilon_{\mathrm{njt}},j=3 \end{array}\right.,$$

where $X_{\mathrm{njt}}$ represents the vector of observable attribute levels related to the COVID‐19 vaccine j; $\beta_{n}^{\prime }$ represents the vector of estimated individual‐specific coefficients; $\varepsilon_{\mathrm{njt}}$ represents the unobserved random term that follows an independently and identically distributed extreme value distribution; $e_{\mathrm{nj}}$ is the error component, which follows the zero‐mean standard normal distribution; $\mu_{n}$ is the coefficient of the estimated error component; and alternative specific constant (*ASC*) is an alternative specific constant representing the opt‐out option, which adopts dummy coding. The opt‐out option is coded as 1; otherwise, it is 0. *ASC* is separately added to the utility of the opt‐out option to capture the potential current situation deviation.^65^ All nonprice attributes adopt effect coding, while the price attribute was coded as a continuous variable. We also tested other forms of the price attribute. The model results can be found in the Appendices S10–S17.

The latent class (LC) model assumes that the choice behaviour of respondents depends on observed variables and latent heterogeneity that the analyst cannot observe. Therefore, the specification divides the population into several classes, each of which has the same preference, and the number of each class is endogenous. Compared with the RPL‐EC model, the LC model assumes that the distribution of coefficients is discrete rather than continuous. Assuming that the population is divided into Q classes, the utility of individual n in class q choosing vaccine j under choice set t is

(2) $$U_{\mathrm{njt}}=\mathrm{ASC}+\beta_{q}^{\prime }X_{\mathrm{njt}}+\varepsilon_{\mathrm{njt}},$$

where $\beta_{q}$ is a class‐specific parameter vector, and the other variables have the same meaning as in Equation (1).

The ECLC model, first proposed by Scarpa et al.,^20^ is an extension of the LC model. Unlike the LC model, the coefficient of the nonattendance attribute in the ECLC model is restricted to 0. There are two kinds of ECLC models in past studies, one of which only considers ANA,^20^, ^25^ which is called the ECLC‐homogeneity model in this paper, and another that considers both ANA and preference heterogeneity,^33^, ^38^ which is called the ECLC‐heterogeneity model in this paper. In the ECLC‐homogeneity model, the coefficients of attendance attributes are restricted to be equal in all classes, which aims to control the influence of preference heterogeneity and ensure that the model focuses only on ANA.^20^, ^66^ The ECLC‐heterogeneity model divides respondents into multiple groups with different preferences, and each group contains different ANA categories. The ECLC‐heterogeneity model constrains the attendance attribute coefficients of all classes to be equal in the same group but allows the change of attendance attribute coefficients between different groups. In this study, the two ECLC models were used to infer the ANA categories of respondents and to factor the impact of ANA into the estimations.

The WTP for COVID‐19 vaccine attributes is calculated as follows^67^:

(3) $$\mathrm{WTP}=-\frac{{2\times \beta }_{\mathrm{nonprice}}}{\beta_{\mathrm{price}}},$$

where $\beta_{\mathrm{nonprice}}$ denotes the coefficient of nonprice attributes and $\beta_{\mathrm{price}}$ denotes the coefficient of price attribute. The 95% confidence intervals for the mean WTP are derived using the Krinsky and Robb^68^ method with 5,000 draws.

## 3 RESULTS

### 3.1 Sociodemographic profiles

A total of 1,576 samples were included in the analyses. The descriptive statistics of the sample (*N *= 1,576) are presented in Table 1. A total of 58.19% of the respondents were female, and the mean age was 33.73 years. A total of 70.43% of the respondents had an average monthly income in 2019 that was no more than $1,233.71 (¥5,000) (the exchange rate used in this paper was from 28 January 2021, which was ¥6.4845 to $1). Slightly more than half of the respondents were married (51.14%) or had no children (54.63%). More than 80% of the respondents had a bachelor's degree or above (83.31%), lived in cities (84.52%), had no chronic diseases (82.80%) and worked in nonmedical‐related industries (93.15%).

**Table 1:** Characteristics of the study sample (*n* = 1,576)

Characteristics	*n*	%
Gender		
Male	659	41.81
Female	917	58.19
Age		
Age 18–25	465	29.51
Age 26–30	318	20.18
Age 31–40	395	25.06
Age 41 or older	398	25.25
Education		
Junior college degree and below	263	16.69
Bachelor's degree	667	42.32
Master's degree and above	646	40.99
Average monthly income in 2019 (unit: $)		
≤308.43 (¥2,000)	398	25.25
308.58–771.07 (¥2,001–¥5,000)	335	21.26
771.22–1,233.71 (¥5,001–¥8,000)	377	23.92
1,233.87–1,850.57 (¥8,001–¥12,000)	248	15.74
≥1,850.72 (≥¥12,001)	218	13.83
Work in a medical‐related industry		
No	1,468	93.15
Yes	108	6.85
Marital status		
Unmarried	770	48.86
Married/divorced/widowed	806	51.14
Children		
No	861	54.63
Yes	715	45.37
Residence		
Rural area	244	15.48
Urban area	1,332	84.52
Chronic disease		
No	1,305	82.80
Yes	271	17.20
Region		
Northeast	178	11.29
North	309	19.61
East	642	40.74
South	221	14.02
Southwest	113	7.17
Northwest	113	7.17

### 3.2 ANA results

There were 2^4^ = 16 kinds of ANA categories. The stepwise approach proposed by Lagarde,^69^ which was used to build the ECLC‐homogeneity model, and the results of the ECLC‐homogeneity model are shown in Table 2 (class memberships), Table 4 (the probability of ANA) and Appendix S3 (model structure). The results showed that only 9.47% of the respondents considered all attributes when making choices, 6.61% of the respondents ignored all attributes and most respondents tended to ignore one attribute (21.21%) or multiple attributes (62.71%). Price was the most often ignored attribute, accounting for 73.30%, followed by the protection period (42.85%), effectiveness (30.15%) and adverse reactions (26.77%).

**Table 2:** Class memberships from the ECLC‐homogeneity model

Class	Description of ANA behaviour	Probability
Class 1	AA (all attendance)	9.47%
Class 2	ANA‐price (only price nonattendance)	21.21%
Class 3	ANA‐effectiveness + price (effectiveness and price nonattendance)	16.92%
Class 4	ANA‐protection period + adverse reactions (protection period and adverse reactions nonattendance)	12.44%
Class 5	ANA‐protection period + price (protection period and price nonattendance)	3.98%
Class 6	ANA‐adverse reactions + price (adverse reactions and price nonattendance)	2.94%
Class 7	AA‐price (only price attendance)	4.79%
Class 8	AA‐adverse reactions (only adverse reactions attendance)	1.83%
Class 9	AA‐effectiveness (only effectiveness attendance)	19.81%
Class 10	ANA (all nonattendance)	6.61%

Abbreviations: ANA, attribute nonattendance; ECLC, equality constraint latent class.

After testing several models with different structures (Appendix S4), an ECLC‐heterogeneity model with the best goodness of fit was obtained. The results are shown in Table 3 (group memberships) and Table 4 (the probability of ANA). The proportions of the four preference groups are 12.92%, 80.33%, 4.75% and 2.00%, respectively. The results showed that 29.09% of the respondents considered all attributes when making choices, 6.77% of them ignored all attributes and price was still the most often ignored attribute, accounting for 64.23%, followed by effectiveness (21.43%), adverse reactions (6.77%) and protection period (6.77%).

**Table 3:** Group memberships from the ECLC‐heterogeneity model

Preference group (probabilities %)	Description of ANA behaviour	Probability
Preference group 1 (12.92%)	AA1 (all attendance)	7.59%
	ANA‐effectiveness1 (only effectiveness nonattendance)	5.33%
Preference group 2 (80.33%)	AA2 (all attendance)	15.82%
	ANA‐price (only price nonattendance)	41.20%
	ANA‐effectiveness2 (only effectiveness nonattendance)	23.31%
Preference group 3 (4.75%)	AA3 (all attendance)	3.60%
	ANA‐effectiveness + price (effectiveness and price nonattendance)	1.15%
Preference group 4 (2.00%)	ANA (all nonattendance)	2.00%

Abbreviations: ANA, attribute nonattendance; ECLC, equality constraint latent class.

**Table 4:** The probability of ANA

	ECLC‐homogeneity model	ECLC‐heterogeneity model
Full attributes attendance	9.47%	29.09%
Effectiveness	30.15%	21.43%
Protection period	42.85%	6.77%
Adverse reactions	26.77%	6.77%
Price	73.30%	64.23%
Full attributes nonattendance	6.61%	6.77%

Abbreviations: ANA, attribute nonattendance; ECLC, equality constraint latent class.

### 3.3 Preferences and WTP for COVID‐19 vaccine attributes

In this paper, RPL‐EC, ECLC‐homogeneity and ECLC‐heterogeneity models were developed. The regression results are shown in Table 5. The goodness of fit for all models was compared using the Bayesian information criteria (BIC) and the Akaike information criteria (AIC). Compared with the RPL‐EC model, the ECLC‐homogeneity model did not significantly improve the goodness of fit; however, the ECLC‐heterogeneity model did improve the fit and obtained the optimal goodness of fit.

**Table 5:** Results of the RPL‐EC, ECLC‐homogeneity and ECLC‐heterogeneity models

			ECLC‐homogeneity model	ECLC‐heterogeneity model		
	RPL‐EC model		AA	AA1	AA2	AA3
Attributes	Coefficient	SD	Coefficient	Coefficient	Coefficient	Coefficient
Vaccine effectiveness						
65% (Controlled level)						
80%	0.522***	0.464***	0.397***	2.903***	0.210**	31.846**
	(0.068)	(0.107)	(0.091)	(0.701)	(0.090)	(15.010)
95%	1.962***	1.960***	2.876***	4.515***	2.831***	−9.541
	(0.110)	(0.130)	(0.159)	(0.663)	(0.137)	(5.881)
Vaccine protection period						
1 year (Controlled level)						
2 years	0.620***	0.032	0.356**	0.806***	0.176*	23.243***
	(0.088)	(1.007)	(0.151)	(0.244)	(0.092)	(6.736)
3 years	0.063	0.520***	0.854***	0.207	0.671***	−45.499***
	(0.100)	(0.124)	(0.191)	(0.198)	(0.114)	(13.277)
Vaccine adverse reactions						
Mild (Controlled level)						
No	1.086***	0.302	1.376***	0.956***	0.821***	27.788**
	(0.076)	(0.214)	(0.141)	(0.142)	(0.055)	(12.270)
Price	−0.002***		−0.013***	−0.002***	−0.008***	−0.136***
			(0.001)	(0.001)	(0.001)	(0.041)
	(0.000)					
*ASC*	−5.566***		−4.854***	3.531***	−4.524***	−97.176***
	(0.340)		(0.514)	(0.668)	(0.297)	(34.730)
Error component		5.031***				
		(0.300)				
Class probability			9.47%	7.59%	15.82%	3.60%
LL	−4,103.057		−4,059.264	−4,022.277		
AIC	8,232.1		8,168.5	8,102.6		
BIC	8,319.8		8,337.3	8,298.3		

*Note*: Standard errors in parentheses.

Abbreviations: AIC, Akaike information criteria; *ASC*, alternative specific constant; BIC, Bayesian information criteria; ECLC, equality constraint latent class; LL, log likelihood; RPL‐EC, random parameter logit model with an error component; SD, standard deviation; SE, standard error.

* *p* < .1

** *p* < .05

*** *p* < .01.

**Figure 2:**
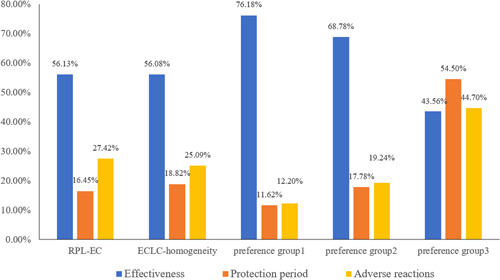
The relative importance of attributes

The relative importance of attributes was estimated, which refers to the difference between the respondents' most preferred level and the least preferred level of each attribute. The greater the relative importance value, the higher the importance of the attribute compared with other attributes, which is determined by the attributes and levels described in this study. The relative importance of attributes was expressed as percentages, and the sum of the percentages of all attributes was equal to 1. The results are shown in Figure 2. Among all models, effectiveness is the most important attribute, followed by adverse reactions and protection period. The exception is preference group 3 in the ECLC‐heterogeneity model, for which the protection period is the most important attribute, followed by adverse reactions and effectiveness.

**Figure 3:**
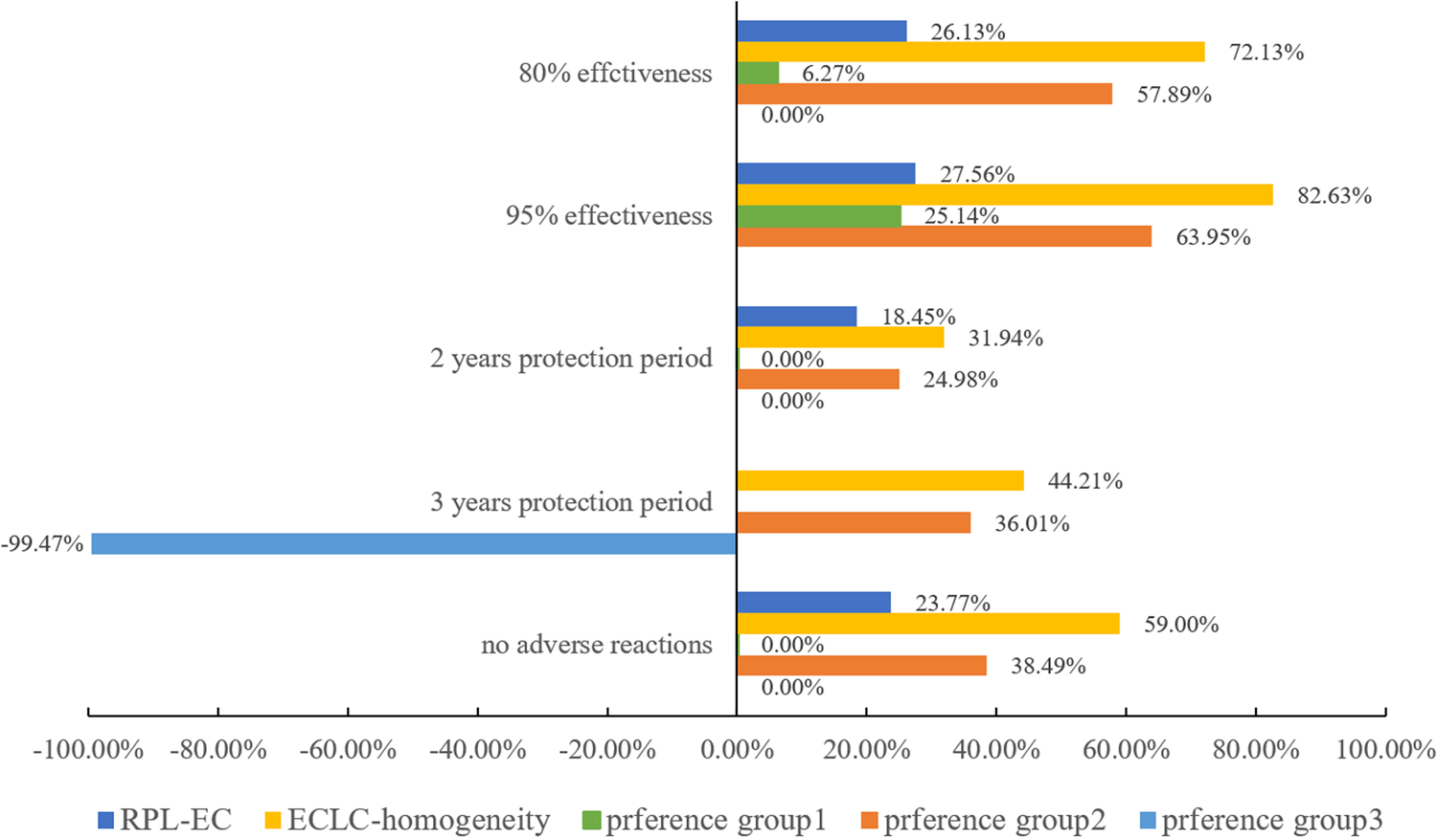
Changes in the prediction probability of vaccination

This paper also predicted the change in the probability of vaccination when only one attribute level changed compared with the base vaccine. For ease of comparison, we defined the base vaccine with 65% effectiveness, a 1‐year protection period, mild adverse reactions and $7.71 (¥50); the results are shown in Figure 3. For attribute levels with insignificant coefficients, we did not calculate the corresponding probability change. The same attribute level was found to have a significantly different impact on the probability of vaccination in different models. In the RPL‐EC model, effectiveness increased from 65% to 80% or 95%, and the probability of vaccination increased by 26.13% and 27.56%. The protection period increased from 1 to 2 years, and the increase was 18.45%. When the adverse reactions changed from mild adverse reactions to no adverse reactions, the increase was 23.77%. For the ECLC‐heterogeneity model, in preference group 1, the probability of vaccination increased by 6.27% and 25.14% when the effectiveness was improved from 65% to 80% or 95%, respectively, and the changes in other attribute levels did not cause changes in vaccination probability. In preference group 2, the effectiveness increased from 65% to 80% or 95%, and the probability of vaccination increased by 57.89% and 63.95%. The protection period increased from 1 to 2 or 3 years, and the improvements were 24.98% and 36.01%, respectively. When the adverse reactions changed from mild adverse reactions to no adverse reactions, the improvement was 38.49%. The results are similar to those of the ECLC‐homogeneity model. In preference group 3, when the protection period was increased from 1 to 3 years, the probability of vaccination decreased by 99.47% (accounting for 4.75% of all respondents), while other attribute levels had almost no significant influence on the probability of vaccination.

In addition, the *χ* ^2^ test was used to test differences in sociodemographic characteristics among the four groups.^70^ The results can be found in Appendix S5. An RPL‐EC model incorporating all sociodemographic characteristics was developed, and the results showed no significant impact on respondents' vaccine choice (Appendix S6). We tested RPL‐EC models with interaction terms and the LC model. The results of all models are shown in Appendix S7 (RPL‐EC models with interaction terms), Appendix S8 (LC model) and Appendix S9 (log likelihood, AIC and BIC information).

The results of WTP are shown in Table 6. Compared with the RPL‐EC model, a lower WTP was obtained in the ECLC‐homogeneity model. The WTP for each attribute level obtained in the RPL‐EC model reached 4.67–11.97 times that in the ECLC‐homogeneous model. Compared with the RPL‐EC model, the WTP in the ECLC‐heterogeneity model yielded mixed results. Preference group 1 had a higher WTP, except for the WTP of adverse reactions. In this group, the WTP for each attribute level was 1.14–4.85 times higher than that in the RPL‐EC model, while adverse reactions were 0.77 times higher. Preference groups 2 and 3 obtained a lower WTP, and the WTP in preference group 2 decreased significantly more than that in preference group 3. The WTP in the RPL‐EC model for each attribute level was 3.07–11.04 times and 1.21–2.88 times the WTP in preference groups 2 and 3 in the ECLC‐heterogeneous model, respectively. Otherwise, in all models, the significant WTP value for 95% effectiveness is the highest, indicating that 95% effectiveness is the highest marginal value among all attribute levels for respondents.

**Table 6:** The WTP results of models ($)^a^

Attributes	RPL‐EC model	ECLC‐homogeneity model	ECLC‐heterogeneity model		
		AA	AA1	AA2	AA3
Vaccine effectiveness					
65% (Controlled level)					
80%	87.00	9.65	422.27	7.88	72.04
	(67.00–109.34)	(5.89–13.34)	(209.38–902.22)	(1.34–15.20)	(13.26–97.18)
95%	327.05	70.02	656.91	106.50	−21.58^b^
	(281.00–383.60)	(58.36–85.37)	(408.90–1298.81)	(92.38–124.29)	(−35.19–7.96)
Vaccine protection period					
1 year (Controlled level)					
2 years	103.30	8.67	117.32	6.62	52.58
	(77.50–130.82)	(1.64–16.34)	(47.32–247.54)	(−0.03–13.92)	(51.01–55.57)
3 years	10.53^b^	20.78	30.11^b^	25.23	−102.93
	(−20.87–45.09)	(11.76–31.34)	(−26.72–110.64)	(17.30–33.89)	(−113.44−95.29)
Vaccine adverse reactions					
Mild (Controlled level)					
No	181.05	33.49	139.05	30.90	62.86
	(154.74–212.38)	(25.69–43.84)	(87.13–262.63)	(25.82–37.47)	(20.31–82.02)

*Note*: 95% confidence intervals (95% CIs) in parentheses.

Abbreviations: ECLC, equality constraint latent class; RPL‐EC, random parameter logit model with an error component; WTP, willingness to pay.

^a^ The exchange rate used in this paper was from 28 January 2021, which was ¥6.4845 to $1.

^b^ The coefficient of the attribute level was not significant.

## 4 DISCUSSION

The ECLC‐heterogeneity model considering both ANA and preference heterogeneity improved the goodness of fit and obtained a lower probability of ANA. The proportion of respondents considering all attributes increased by 19.82%, and the proportion of respondents ignoring effectiveness, protection period, adverse reactions and price decreased by 8.72%, 36.08%, 20.00% and 9.07%, respectively, indicating that the probability of ANA of all attributes decreased when preference heterogeneity was considered.^24^, ^71^, ^72^ This result supports the discovery of confusion between ANA and preference heterogeneity in previous studies, which is explained by the fact that the ECLC‐homogeneity model may fail to distinguish between real nonattendance and low preference, and some respondents with low preference are identified as nonattenders, resulting in the overestimation of ANA.^24^, ^27^ In addition, although the probability of ANA was lower in the ECLC‐heterogeneity model than in the ECLC‐homogeneity model, the probability of ANA was still high (29.09% of respondents considered all attributes, and 64.23% of respondents ignored price), which shows that considering preference heterogeneity could not completely accommodate the influence of ANA.^24^ In other words, some respondents in this study may truly ignore some attributes rather than giving them low importance.

Compared with the RPL‐EC model, the two ECLC models considering ANA obtained inconsistent WTP estimates. Lower WTP was obtained in the ECLC‐homogeneity model, which is similar to some previous studies.^35^, ^73^ Mixed WTP results were obtained in the ECLC‐heterogeneity model, with preference group 1 having a higher WTP (only the WTP for no adverse reactions decreased). Preference groups 2 and 3 had a lower WTP, which has also been found in some previous studies.^38^, ^74^ For example, in a study of four rural landscape improvement choices in the Republic of Ireland, the LC model considering both ANA and preference heterogeneity also obtained higher and/or lower WTP results compared with the model without ANA.^74^ Caputo et al.^38^ studied consumer preferences for two labels of the food transportation footprint and divided respondents into two preference groups. It was found that compared with the RPL‐EC model, preference group 1 obtained lower WTP estimation, while preference group 2 obtained higher WTP estimation. The results of this paper indicated that regardless of whether we consider only ANA or both ANA and preference heterogeneity, all considerations affected the WTP estimates.

Because ANA is an empirical problem, whether each study needs to consider ANA depends on the situation. Future studies may need to pay more attention to ANA in the following three situations. First, ANA should be considered when respondents are unfamiliar with the research object or the choice sets are complex because people are more likely to adopt simplified heuristics in such cases,^27^ which may have happened in this study. Second, when incredible coefficient symbols appear in the model, this result may be caused by failure to take ANA into account because studies have shown that failure to consider ANA may lead to unusual symbols of random parameter coefficients.^75^ Finally, the results of the stated ANA can be used as a predictor of ANA; it has been proven in some studies that stated ANA is an effective indicator of the probability of ANA.^71^ In the future, researchers could pay more attention to ANA under different circumstances and to investigate what factors may lead to a high probability of ANA, such as whether the levels of one attribute are two times or more times the levels of other attributes, whether the number of choice sets will have significant impacts on ANA results, and so forth.

## 5 CONCLUSIONS

This study examined Chinese public preferences for COVID‐19 vaccine attributes and their WTP using DCE by accounting for ANA. Effectiveness was the most important attribute, followed by adverse reactions and protection period. The RPL‐EC model considered only preference heterogeneity, the ECLC‐homogeneity model considered only ANA and the ECLC‐heterogeneity model considered both. The three models obtained inconsistent WTP for COVID‐19 vaccine attributes. The results of the ECLC‐heterogeneity model considering both ANA and preference heterogeneity are more plausible because ANA and low preference may be confounded in the ECLC‐homogeneity model considering only ANA and the PRL‐EC model considering only preference heterogeneity. Although the probability of ANA was lower in the ECLC‐heterogeneity model than in the ECLC‐homogeneity model, there was still a high probability of ANA. Therefore, in future research on DCE (such as the field of vaccines), ANA is an essential issue that should be considered.

## AUTHOR CONTRIBUTIONS

Jianhong Xiao contributed to the overall research design and paper writing; Fei Wang analysed and interpreted the data, and contributed to paper writing; Min Wang performed data collection and analysed the data; and Zegang Ma contributed to questionnaire design and writing of the paper.

## CONFLICT OF INTERESTS

Authors declare no conflict of interests.

## Supporting information

Supplementary information.

Supplementary information.

## Data Availability

The data that support the findings of this study are available from the corresponding author upon reasonable request.
