# Supplementary material for: Attribute nonattendance in COVID‐19 vaccine choice: A discrete choice experiment based on Chinese public preference
Source: Health Expect. 2022 Jan 20;25(3):959–70. doi: 10.1111/hex.13439 (PMC9122444; doi:10.1111/hex.13439)
Supplement: Supplementary file 1 — Supplementary information. [file HEX-25--s001.docx]

Appendix Table 1 Attributes and levels of the COVID-19 vaccine

| Attribute | Levels |
| --- | --- |
| Effectiveness | 65% |
|  | 80% |
|  | 95% |
| Protection period | 1 year |
|  | 2 years |
|  | 3 years |
| Adverse reactions | Mild adverse reactions |
|  | No adverse reactions |
| Price (Unit: $) | 7.71 (¥50) |
|  | 15.42 (¥100) |
|  | 30.84 (¥200) |
|  | 46.26 (¥300) |
|  | 77.11 (¥500) |
|  | 154.21 (¥1,000) |

Appendix Table 2 Sample distribution and snowball sampling

| Region ^a^ | Two hundred seventeen recruited persons percentage ^b^ | The final sample percentage | Total population percentage ^c^ |
| --- | --- | --- | --- |
| North | 16.13% | 19.61% | 11.99% |
| Northeast | 4.61% | 11.29% | 6.98% |
| East | 35.48% | 40.74% | 30.00% |
| South | 23.50% | 14.02% | 29.03% |
| Southwest | 12.44% | 7.17% | 14.53% |
| Northwest | 7.83% | 7.17% | 7.33% |

Notes: a. The North region includes Beijing, Tianjin, Hebei Province, Shanxi Province and Inner Mongolia Autonomous Region; the Northeast region includes Liaoning Province, Jilin Province and Heilongjiang Province; the East region includes Shanghai, Jiangsu Province, Zhejiang Province, Anhui Province, Fujian Province, Jiangxi Province and Shandong Province; the South region includes Henan Province, Hubei Province, Hunan Province, Guangdong Province, Guangxi Province and Hainan Province; the Southwest region includes Chongqing, Sichuan Province, Guizhou Province, Yunnan Province and Tibet Autonomous Region; and the Northwest region includes Shaanxi Province, Gansu Province, Qinghai Province, Ningxia Hui Autonomous Region and Xinjiang Uygur Autonomous Region. b. A total of 217 people from 31 provinces in mainland China were recruited by the authors, and they were asked to continue snowballing and sending questionnaires. c. The percentage of the population in each region was calculated based on data from the National Bureau of Statistics (http://www.stats.gov.cn/tjsj/tjgb/rkpcgb/qgrkpcgb/202106/t20210628_1818822.html).

Appendix Table 3 The model structure and class probability of the ECLC-homogeneity model

| Class | Coefficient | | | | | | | Model1 | Model2 | Model3 | Model4 | Model-final |
| --- | --- | --- | --- | --- | --- | --- | --- | --- | --- | --- | --- | --- |
| 1 | $\beta_{eff2}$ | $\beta_{eff3}$ | $\beta_{pro2}$ | $\beta_{pro3}$ | $\beta_{side2}$ | $\beta_{price}$ | $\beta_{ASC1}$ | 0.105^***^ | 0.133^***^ | 0.113^***^ | 0.093^***^ | 0.095^***^ |
| 2 | $0$ | $0$ | $\beta_{pro2}$ | $\beta_{pro3}$ | $\beta_{side2}$ | $\beta_{price}$ | $\beta_{ASC2}$ | 0.285^***^ | 0.032^***^ | 0.000 |  |  |
| 3 | $\beta_{eff2}$ | $\beta_{eff3}$ | $0$ | $0$ | $\beta_{side2}$ | $\beta_{price}$ | $\beta_{ASC3}$ | 0.045^***^ | 0.051 |  |  |  |
| 4 | $\beta_{eff2}$ | $\beta_{eff3}$ | $\beta_{pro2}$ | $\beta_{pro3}$ | $0$ | $\beta_{price}$ | $\beta_{ASC4}$ | 0.053 |  |  |  |  |
| 5 | $\beta_{eff2}$ | $\beta_{eff3}$ | $\beta_{pro2}$ | $\beta_{pro3}$ | $\beta_{side2}$ | $0$ | $\beta_{ASC5}$ | 0.511^***^ | 0.285^***^ | 0.226^***^ | 0.212^***^ | 0.212^***^ |
| 6 | $0$ | $0$ | $0$ | $0$ | $\beta_{side2}$ | $\beta_{price}$ | $\beta_{ASC6}$ |  | 0.000 |  |  |  |
| 7 | $0$ | $0$ | $\beta_{pro2}$ | $\beta_{pro3}$ | $0$ | $\beta_{price}$ | $\beta_{ASC7}$ |  | 0.030 |  |  |  |
| 8 | $0$ | $0$ | $\beta_{pro2}$ | $\beta_{pro3}$ | $\beta_{side2}$ | $0$ | $\beta_{ASC8}$ |  | 0.197^***^ | 0.169^***^ | 0.168^***^ | 0.169^***^ |
| 9 | $\beta_{eff2}$ | $\beta_{eff3}$ | $0$ | $0$ | $0$ | $\beta_{price}$ | $\beta_{ASC9}$ |  | 0.098^***^ | 0.123^***^ | 0.125^***^ | 0.124^***^ |
| 10 | $\beta_{eff2}$ | $\beta_{eff3}$ | $0$ | $0$ | $\beta_{side2}$ | $0$ | $\beta_{ASC10}$ |  | 0.051^***^ | 0.033^**^ | 0.040^***^ | 0.040^***^ |
| 11 | $\beta_{eff2}$ | $\beta_{eff3}$ | $\beta_{pro2}$ | $\beta_{pro3}$ | $0$ | $0$ | $\beta_{ASC11}$ |  | 0.122^***^ | 0.025^***^ | 0.029^***^ | 0.029^***^ |
| 12 | $0$ | $0$ | $0$ | $0$ | $0$ | $\beta_{price}$ | $\beta_{ASC12}$ |  |  | 0.038^***^ | 0.048^***^ | 0.048^***^ |
| 13 | $0$ | $0$ | $0$ | $0$ | $\beta_{side2}$ | $0$ | $\beta_{ASC13}$ |  |  | 0.018^**^ | 0.018^***^ | 0.018^***^ |
| 14 | $0$ | $0$ | $\beta_{pro2}$ | $\beta_{pro3}$ | $0$ | $0$ | $\beta_{ASC14}$ |  |  | 0.060 |  |  |
| 15 | $\beta_{eff2}$ | $\beta_{eff3}$ | $0$ | $0$ | $0$ | $0$ | $\beta_{ASC15}$ |  |  | 0.139^***^ | 0.199^***^ | 0.198^***^ |
| 16 | $0$ | $0$ | $0$ | $0$ | $0$ | $0$ | $\beta_{ASC16}$ |  |  | 0.056^***^ | 0.063^***^ | 0.066^***^ |
| 17 | $0$ | $0$ | $0$ | $0$ | $0$ | $0$ | $0$ |  |  |  | 0.005 |  |
| LL |  |  |  |  |  |  |  | -4,135.037 | -4,075.898 | -4,043.907 | -4,058.967 | -4,059.264 |
| AIC |  |  |  |  |  |  |  | 8,300.1 | 8,201.8 | 8,145.8 | 8,169.9 | 8,168.5 |
| BIC |  |  |  |  |  |  |  | 8,401.3 | 8,370.5 | 8,341.5 | 8,345.4 | 8,337.3 |

Notes: (1) $\beta_{eff2}$ and $\beta_{eff3}$ indicate the coefficients of 80% effectiveness and 95% effectiveness, respectively; $\beta_{pro2}$ and $\beta_{pro3}$ indicate the coefficients of the 2-year protection period and 3-year protection period, respectively; $\beta_{side2}$ indicates the coefficient of no adverse reactions; $\beta_{price}$ indicates the coefficient of price; $\beta_{ASCq}$ indicates the coefficient of *ASC* in class $q$; (2) ^*^p<0.1, ^**^p<0.05, ^***^p<0.01.

Appendix Table 4 Information on the convergent models in the tested ECLC-heterogeneity models

| Model number | Specification | Number of classes | LL | AIC | BIC |
| --- | --- | --- | --- | --- | --- |
| 1 | Group 1 (AA1+ANA-price)+Group 2 (AA2) | 3 | -4,447.233 | 8,926.5 | 9,034.4 |
| 2 | Group 1 (AA1+ANA-eff)+Group 2 (AA2) | 3 | -4,202.292 | 8,436.6 | 8,544.6 |
| 3 | Group 1 (AA1+ANA-eff)+Group 2 (AA2+ANA-price) | 4 | -4,187.219 | 8,408.4 | 8,523.2 |
| 4 | Group 1 (AA1+ANA-eff)+Group 2 (AA2+ANA-side) | 4 | -4,178.473 | 8,390.9 | 8,505.7 |
| 5 | Group 1 (AA1+ANA-side)+Group 2 (AA2+ANA-price) | 4 | -4,235.478 | 8,505.0 | 8,619.7 |
| 6 | Group 1 (AA1+ANA-eff)+Group 2 (AA2+ANA-price+ANA) | 5 | -4,255.370 | 8,546.7 | 8,668.2 |
| 7 | Group 1 (AA1+ANA-eff+ANA)+Group 2 (AA2+ANA-pro) | 5 | -4,283.231 | 8,602.5 | 8,723.9 |
| 8 | Group 1 (AA1+ANA-eff+ANA-eff/side)+Group 2 (AA2+ANA-side) | 5 | -4,170.719 | 8,377.4 | 8,498.9 |
| 9 | Group 1 (AA1+ANA-eff+ANA-price)+Group 2 (AA2+ANA-pro/price) | 5 | -4,171.614 | 8,379.2 | 8,500.7 |
| 10 | Group 1 (AA1+ANA-eff)+Group 2 (AA2+ANA-price+ANA-eff/price) | 5 | -4,093.171 | 8,222.3 | 8,343.8 |
| 11 | Group 1 (AA1+ANA-pro)+Group 2 (AA2+ANA-side+ANA-eff/side) | 5 | -4,194.868 | 8,425.7 | 8,547.2 |
| 12 | Group 1 (AA1+ANA-side+ANA-side/price)+Group 2 (AA2+ANA-price) | 5 | -4,228.609 | 8,493.2 | 8,614.7 |
| 13 | Group 1 (AA1+ANA-eff+ANA-side+ANA)+Group 2 (AA2+ANA-eff/side) | 6 | -4,089.468 | 8,216.9 | 8,345.2 |
| 14 | Group 1 (AA1+ANA-eff+ANA-side)+Group 2 (AA2+ANA-eff/side)+Group 3 (ANA) | 6 | -4,074.164 | 8,188.3 | 8,323.3 |
| 15 | Group 1 (AA1+ANA-eff)+Group 2 (AA2+ANA-price+ANA-eff/price+ANA) | 6 | -4,083.069 | 8,204.1 | 8,332.4 |
| 16 | Group 1 (AA1+ANA-eff)+Group 2 (AA2+ANA-price+ANA-eff/price)+Group 3 (ANA) | 6 | -4,069.333 | 8,178.7 | 8,313.6 |
| 17 | Group 1 (AA1+ANA-eff+ANA-side/pro/price)+Group 2 (AA2+ANA-price+ANA-eff/price)+Group 3 (ANA) | 7 | -4,067.443 | 8,176.9 | 8,318.6 |
| 18 | Group 1 (AA1+ANA-side/pro/price+ANA-pro/price)+Group 2 (AA2+ANA-price+ANA-eff/price)+Group 3 (ANA) | 7 | -4,137.067 | 8,316.1 | 8,457.9 |
| 19 | Group 1 (AA1+ANA-eff)+Group 2 (AA2+ANA-price+ANA-eff/price)+Group 3 (AA3+ANA-eff/price)+ANA | 8 | -4,022.277 | 8,102.6 | 8,298.3 |
| 20 | Group 1 (AA1+ANA-eff)+Group 2 (AA2+ANA-price+ANA-eff/price)+Group 3 (AA3+ANA-pro/price+ANA) | 8 | -4,090.739 | 8,237.5 | 8,426.4 |
| 21 | Group 1 (AA1+ANA-eff)+Group 2 (AA2+ANA-price+ANA-eff/price)+Group 3 (AA3+ANA-eff/price+ANA-pro/price)+ANA | 9 | -4,028.123 | 8,116.2 | 8,318.7 |

Notes: AA1 means the class in preference group 1 exhibited attendance to all attributes; ANA-price means the class is only price nonattendance; and ANA-eff/side means the class is effectiveness and adverse reactions nonattendance. The remaining variables are similarly defined.

Appendix Table 5 Characteristics of respondents from different preference groups in the ECLC-heterogeneity model

| Characteristics | Preference group 1 n=217 | Preference  group 2 n=1,285 | Preference group 3 n=55 | Preference group 4 n=19 | χ2 statistic  (P-value) |
| --- | --- | --- | --- | --- | --- |
| Gender |  |  |  |  |  |
| Male | 39.17% | 42.26% | 43.64% | 36.84% | 0.995  (0.802) |
| Female | 60.83% | 57.74% | 56.36% | 63.16% |  |
| Age |  |  |  |  |  |
| Age 18-25 | 24.42% | 30.19% | 40.00% | 10.53% | 27.036  (0.001) |
| Age 26-30 | 19.82% | 19.69% | 20.00% | 57.89% |  |
| Age 31-40 | 25.35% | 25.68% | 14.55% | 10.53% |  |
| Age 41 and older | 30.41% | 24.44% | 25.45% | 21.05% |  |
| Education |  |  |  |  |  |
| Junior college degree and below | 21.66% | 15.64% | 21.82% | 15.79% | 6.570  (0.362) |
| Bachelor’s degree | 39.17% | 42.72% | 43.64% | 47.37% |  |
| Master’s degree and above | 39.17% | 41.63% | 34.55% | 36.84% |  |
| Average monthly income in 2019 (unit: $) | | | | | |
| ≤308.43 (¥2,000) | 17.97% | 25.68% | 40.00% | 36.84% | 25.468  (0.013) |
| 308.58-771.07 (¥2,001-¥5,000) | 27.19% | 20.31% | 25.45% | 5.26% |  |
| 771.22-1,233.71 (¥5,001-¥8,000) | 23.50% | 24.36% | 14.55% | 26.32% |  |
| 1,233.87-1,850.57  (¥8,001-¥12,000) | 18.43% | 15.25% | 12.73% | 26.32% |  |
| ≥1,850.72 (≥¥12,001) | 12.90% | 14.40% | 7.27% | 5.26% |  |
| Work in a medical-related industry |  |  |  |  |  |
| No | 93.55% | 92.84% | 96.36% | 100.00% | 2.533  (0.469) |
| Yes | 6.45% | 7.16% | 3.64% | 0.00% |  |
| Marital status |  |  |  |  |  |
| Unmarried | 39.17% | 50.04% | 54.55% | 63.16% | 11.134  (0.011) |
| Married/divorced/widowed | 60.83% | 49.96% | 45.45% | 36.84% |  |
| Children |  |  |  |  |  |
| No | 52.07% | 44.59% | 40.00% | 36.84% | 5.446  (0.142) |
| Yes | 47.93% | 55.41% | 60.00% | 63.16% |  |
| Residence |  |  |  |  |  |
| Rural area | 87.56% | 84.82% | 69.09% | 73.68% | 13.332  (0.004) |
| Urban area | 12.44% | 15.18% | 30.91% | 26.32% |  |
| Chronic disease |  |  |  |  |  |
| No | 19.35% | 17.04% | 9.09% | 26.32% | 4.379  (0.223) |
| Yes | 80.65% | 82.96% | 90.91% | 73.68% |  |
| Region |  |  |  |  |  |
| Northeast | 12.90% | 10.82% | 10.91% | 26.32% | 20.399  (0.157) |
| North | 22.12% | 19.30% | 18.18% | 15.79% |  |
| East | 37.79% | 41.40% | 41.82% | 26.32% |  |
| South | 11.52% | 14.09% | 21.82% | 15.79% |  |
| Southwest | 5.53% | 7.63% | 5.45% | 0.00% |  |
| Northwest | 10.14% | 6.77% | 1.82% | 15.79% |  |

Appendix Table 6 The results of the RPL model and RPL-EC model with sociodemographic characteristics

| Attribute | RPL Model | | RPL-EC Model  (with sociodemographic characteristics) | |
| --- | --- | --- | --- | --- |
|  | Coefficient | SD | Coefficient | SD |
| Vaccine effectiveness | | | | |
| 65% (Controlled level) | | | | |
| 80% | 0.397^***^  (0.040) | 0.057  (0.389) | 0.522^***^  (0.068) | 0.462^***^  (0.106) |
| 95% | 1.287^***^  (0.055) | 1.370^***^  (0.063) | 1.962^***^  (0.110) | 1.964^***^  (0.130) |
| Vaccine protection period | | | | |
| 1 year (Controlled level) | | | | |
| 2 years | 0.345^***^  (0.047) | 0.001  (0.067) | 0.619^***^  (0.088) | 0.025  (1.115) |
| 3 years | 0.088^*^  (0.053) | 0.001  (0.093) | 0.064  (0.100) | 0.522^***^  (0.124) |
| Vaccine adverse reactions | | | | |
| Mild (Controlled level) | | | | |
| No | 0.684^***^  (0.040) | 0.001  (0.074) | 1.083^***^  (0.076) | 0.292  (0.220) |
| Price | -0.001^***^  (0.000) |  | -0.002^***^  (0.000) |  |
| *ASC* | -1.564^***^  (0.059) |  | -4.787^***^  (0.887) |  |
| *ASC**age1 |  |  |  |  |
| *ASC**age2 |  |  | 0.007  (0.606) |  |
| *ASC**age3 |  |  | -0.982  (0.799) |  |
| *ASC**age4 |  |  | -0.610  (0.867) |  |
| *ASC**gender |  |  | -0.194  (0.359) |  |
| *ASC**education1 |  |  |  |  |
| *ASC**education2 |  |  | -0.309  (0.515) |  |
| *ASC**education3 |  |  | -0.169  (0.536) |  |
| *ASC**average monthly income1 in 2019 |  |  |  |  |
| *ASC**average monthly income2 in 2019 |  |  | 0.132  (0.585) |  |
| *ASC**average monthly income3 in 2019 |  |  | -0.028  (0.646) |  |
| *ASC**average monthly income4 in 2019 |  |  | 0.778  (0.696) |  |
| *ASC**average monthly income5 in 2019 |  |  | -0.390  (0.745) |  |
| *ASC**work in a medical-related industry |  |  | -0.884  (0.715) |  |
| *ASC**marital status |  |  | 1.368  (0.838) |  |
| *ASC**children |  |  | -0.382  (0.804) |  |
| *ASC**region1 |  |  |  |  |
| *ASC**region2 |  |  | -0.550  (0.651) |  |
| *ASC**region3 |  |  | -1.008^*^  (0.587) |  |
| *ASC**region4 |  |  | -1.382^*^  (0.731) |  |
| *ASC**region5 |  |  | -1.247  (0.856) |  |
| *ASC**region6 |  |  | 0.345  (0.786) |  |
| *ASC**residence |  |  | 0.073  (0.539) |  |
| *ASC**chronic disease |  |  | 0.449  (0.463) |  |
| Error component |  |  |  | -4.962^***^  (0.301) |
| LL | -4,926.039 |  | -4,091.542 |  |
| AIC | 9,876.1 |  | 8,249.1 |  |
| BIC | 9,957.1 |  | 8,471.8 |  |

Notes: (1) SD means standard deviation; (2) Standard errors (SE) in parentheses; (3) ^*^p<0.1, ^**^p<0.05, ^***^p<0.01.

Appendix Table 7 The results of the RPL-EC model with interaction terms

| Attribute | RPL-EC model with effectiveness*protection | | RPL-EC model with effectiveness*adverse | | RPL-EC model with protection*adverse | |
| --- | --- | --- | --- | --- | --- | --- |
|  | Coefficient | SD | Coefficient | SD | Coefficient | SD |
| Vaccine effectiveness | | | | | | |
| 65% (Controlled level) | | | | | | |
| 80% | 0.459^***^  (0.071) | 0.434^***^  (0.113) | 0.388^***^  (0.093) | 0.473^***^  (0.109) | 0.668^***^  (0.096) | 0.491^***^  (0.105) |
| 95% | 1.875^***^  (0.114) | 1.870^***^  (0.131) | 2.018^***^  (0.116) | 1.944^***^  (0.134) | 1.819^***^  (0.148) | 2.029^***^  (0.128) |
| Vaccine protection period | | | | | | |
| 1 year (Controlled level) | | | | | | |
| 2 years | 0.445^***^  (0.101) | 0.015  (1.007) | 0.450^***^  (0.130) | 0.036  (0.928) | 0.685^***^  (0.116) | 0.007  (1.281) |
| 3 years | 0.140  (0.111) | 0.405^***^  (0.146) | 0.202  (0.131) | 0.528^***^  (0.124) | -0.070  (0.133) | 0.567^***^  (0.118) |
| Vaccine adverse reactions | | | | | | |
| Mild (Controlled level) | | | | | | |
| No | 0.806^***^  (0.085) | 0.342^*^  (0.194) | 0.989^***^  (0.100) | 0.439^**^  (0.174) | 1.141^***^  (0.079) | 0.170  (0.373) |
| Price | -0.002^***^  (0.000) |  | -0.002^***^  (0.000) |  | -0.002^***^  (0.000) |  |
| *ASC* | -5.899^***^  (0.383) |  | -5.410^***^  (0.356) |  | -5.551^***^  (0.350) |  |
| eff2*pro2 | 0.108  (0.113) |  |  |  |  |  |
| eff3*pro2 | 0.440^***^  (0.118) |  |  |  |  |  |
| eff2*pro3 | 0.526^***^  (0.097) |  |  |  |  |  |
| eff3*pro3 | -0.775^***^  (0.171) |  |  |  |  |  |
| eff2*side2 |  |  | -0.181^*^  (0.098) |  |  |  |
| eff3*side2 |  |  | 0.328^**^  (0.163) |  |  |  |
| pro2*side2 |  |  |  |  | 0.013  (0.085) |  |
| pro3*side2 |  |  |  |  | -0.186^*^  (0.110) |  |
| Error component |  | -5.376^***^  (0.337) |  | -4.971^***^  (0.305) |  | -5.044^***^  (0.311) |
| LL | -4,074.269 |  | -4,101.066 |  | -4,098.370 |  |
| AIC | 8,182.5 |  | 8,232.1 |  | 8,226.7 |  |
| BIC | 8,297.3 |  | 8,333.4 |  | 8,328.0 |  |

Notes: (1) SD means standard deviation; (2) Standard errors (SE) in parentheses; (3) ^*^p<0.1, ^**^p<0.05, ^***^p<0.01; (4) eff2 and eff3 are the coefficients of 80% effectiveness and 95% effectiveness, respectively; pro2 and pro3 are the coefficients of 2-year protection period and 3-year protection period, respectively; side2 is the coefficient of no adverse reactions.

Appendix table 8 The results of the LC model with 2 classes

| Attribute | LC model (2 classes) | | | |
| --- | --- | --- | --- | --- |
|  | Class1 | | Class2 | |
|  | Coefficient | SE | Coefficient | SE |
| Vaccine effectiveness |  |  |  |  |
| 65% (Controlled level) | | | | |
| 80% | 0.270^***^ | 0.048 | 0.552^***^ | 0.202 |
| 95% | 1.092^***^ | 0.038 | 1.590^***^ | 0.196 |
| Vaccine protection period |  |  |  |  |
| 1 year (Controlled level) | | | | |
| 2 years | 0.437^***^ | 0.068 | 0.379^***^ | 0.134 |
| 3 years | 0.011 | 0.083 | 0.194 | 0.143 |
| Vaccine adverse reactions |  |  |  |  |
| Mild (Controlled level) | | | | |
| No | 0.741^***^ | 0.047 | 0.860^***^ | 0.106 |
| Price | -0.001^***^ | 0.000 | -0.002^***^ | 0.001 |
| *ASC* | -3.082^***^ | 0.105 | 1.816^***^ | 0.231 |
| Class probability | 0.865^***^ |  | 0.135^***^ |  |
| LL | -4,472.082 |  |  |  |
| AIC | 8,974.2 |  |  |  |
| BIC | 9,075.4 |  |  |  |

Notes: (1) SE means standard errors; (2) ^*^p<0.1, ^**^p<0.05, ^***^p<0.01.

Appendix table 9 The LL, AIC, BIC information of models

| Model | LL | AIC | BIC |
| --- | --- | --- | --- |
| RPL Model | -4,926.039 | 9,876.1 | 9,957.1 |
| RPL-EC Model | -4,103.057 | 8,232.1 | 8,319.8 |
| RPL-EC model (with sociodemographic characteristics) | -4,091.542 | 8,249.1 | 8,471.8 |
| RPL-EC model with effectiveness*protection | -4,074.269 | 8,182.5 | 8,297.3 |
| RPL-EC model with effectiveness*adverse | -4,101.066 | 8,232.1 | 8,333.4 |
| RPL-EC model with protection*adverse | -4,098.370 | 8,226.7 | 8,328.0 |
| Latent class model (2 classes) | -4,472.082 | 8,974.2 | 9,075.4 |
| RPL model in WTP space | -4,600.543 | 9,229.1 | 9,323.6 |
| Nested logit model | -5,175.638 | 10,367.3 | 10,421.3 |
| ECLC-homogeneity model | -4,059.264 | 8,168.5 | 8,337.3 |
| ECLC-heterogeneity model | -4,022.277 | 8,102.6 | 8,298.3 |

Notes: (1) RPL-EC model (with sociodemographic characteristics) means that we added all sociodemographic characteristics in the RPL-EC model; (3) RPL-EC model with effectiveness*protection means the RPL-EC model with an interaction term of effectiveness and protection period; (4) RPL-EC model with effectiveness*adverse means the RPL-EC model with an interaction term of effectiveness and adverse reactions; (5) RPL-EC model with protection*adverse means the RPL-EC model with an interaction term of protection period and adverse reactions; (6) ECLC-homogeneity model means the ECLC model which only consider ANA and assumed preference homogeneity; and (7) ECLC-heterogeneity model means the ECLC model which considered both ANA and preference heterogeneity.

Appendix table 10 The results of the RPL model with (price^2)/100

| Attribute | RPL model with (price^2)/100 | |
| --- | --- | --- |
|  | Coefficient | SD |
| Vaccine effectiveness | | |
| 65% (Controlled level) | | |
| 80% | 0.381^***^  (0.040) | 0.144  (0.172) |
| 95% | 1.269^***^  (0.057) | 1.427^***^  (0.067) |
| Vaccine protection period | | |
| 1 year (Controlled level) | | |
| 2 years | 0.349^***^  (0.046) | 0.001  (0.069) |
| 3 years | 0.018  (0.053) | 0.001  (0.109) |
| Vaccine adverse reactions | | |
| Mild (Controlled level) | | |
| No | 0.582^***^  (0.043) | 0.001  (0.082) |
| Price | 0.002^***^  (0.000) |  |
| Price^2/100 | -0.000^***^  (0.000) |  |
| *ASC* | -1.103^***^  (0.088) |  |
| LL | -4,901.260 |  |
| AIC | 9,828.5 |  |
| BIC | 9,916.3 |  |

Notes: (1) SD means standard deviation; (2) Standard errors (SE) in parentheses; (3) ^*^p<0.1, ^**^p<0.05, ^***^p<0.01.

Appendix table 11 The results of the RPL, RPL-EC and LC models (price adopts effect coding)

| Attribute | RPL model | | RPL-EC model | | LC model | |
| --- | --- | --- | --- | --- | --- | --- |
|  | Coefficient | SD | Coefficient | SD | Class 1 | Class2 |
| Vaccine effectiveness | | | | | | |
| 65% (Controlled level) | | | | | | |
| 80% | 0.443^***^  (0.042) | 0.255^**^  (0.109) | 0.582^***^  (0.075) | 0.513^***^  (0.107) | 0.327^***^  (0.061) | 0.469^**^  (0.221) |
| 95% | 1.046^***^  (0.065) | 1.492^***^  (0.069) | 1.834^***^  (0.129) | 2.001^***^  (0.138) | 1.510^***^  (0.099) | 0.130  (0.105) |
| Vaccine protection period | | | | | | |
| 1 year (Controlled level) | | | | | | |
| 2 years | 0.410^***^  (0.047) | 0.001  (0.067) | 0.693^***^  (0.092) | 0.028  (1.016) | 0.524^***^  (0.085) | 0.438^**^  (0.195) |
| 3 years | -0.181^***^  (0.059) | 0.005  (0.166) | -0.193  (0.129) | 0.568^***^  (0.121) | -0.210  (0.131) | 0.677^***^  (0.142) |
| Vaccine adverse reactions | | | | | | |
| Mild (Controlled level) | | | | | | |
| No | 0.611^***^  (0.045) | 0.002  (0.090) | 1.033^***^  (0.082) | 0.309  (0.231) | 0.562^***^  (0.063) | 1.714^***^  (0.195) |
| Price1 (Controlled level) | | | | | | |
| Price2 | 0.294^***^  (0.081) |  | 0.136  (0.164) |  | -0.663^***^  (0.191) | 1.302^***^  (0.224) |
| Price3 | 0.044  (0.064) |  | 0.294^**^  (0.117) |  | 0.574^***^  (0.133) | -0.304  (0.222) |
| Price4 | 0.837^***^  (0.082) |  | 0.883^***^  (0.180) |  | 0.711^***^  (0.181) | 0.562^*^  (0.288) |
| Price5 | -0.038  (0.058) |  | -0.335^***^  (0.094) |  | -0.302^***^  (0.090) | -1.116^***^  (0.378) |
| Price6 | -0.752^***^  (0.067) |  | -1.506^***^  (0.141) |  | -1.331^***^  (0.174) | -0.423^*^  (0.219) |
| *ASC* | -1.081^***^  (0.049) |  | -4.934^***^  (0.343) |  | -3.202^***^  (0.147) | 1.176^***^  (0.175) |
| Class probability |  |  |  |  | 0.766^***^ | 0.234^***^ |
| Error component |  |  | -5.110^***^  (0.321) |  |  |  |
| LL | -4,862.250 |  | -4,083.806 |  | -4,508.384 |  |
| AIC | 9,756.5 |  | 8,201.6 |  | 9,062.8 |  |
| BIC | 9,864.5 |  | 8,316.3 |  | 9,218.0 |  |

Notes: (1) SD means standard deviation; (2) Standard errors (SE) in parentheses; (3) ^*^p<0.1, ^**^p<0.05, ^***^p<0.01.

Appendix table 12 The results of the RPL-EC model with sociodemographic characteristics (price adopts effect coding)

| Attribute | RPL-EC Model  (with sociodemographic characteristics) | |
| --- | --- | --- |
|  | Coefficient | SD |
| Vaccine effectiveness | | |
| 65% (Controlled level) |  |  |
| 80% | 0.581^***^  (0.076) | 0.508^***^  (0.106) |
| 95% | 1.817^***^  (0.127) | 2.010^***^  (0.134) |
| Vaccine protection period | | |
| 1 year (Controlled level) |  |  |
| 2 years | 0.684^***^  (0.092) | 0.008  (1.119) |
| 3 years | -0.186  (0.129) | 0.552^***^  (0.123) |
| Vaccine adverse reactions | | |
| Mild (Controlled level) |  |  |
| No | 1.024^***^  (0.083) | 0.228  (0.287) |
| Price1 (Controlled level) |  |  |
| Price2 | 0.147  (0.165) |  |
| Price3 | 0.287^**^  (0.119) |  |
| Price4 | 0.872^***^  (0.184) |  |
| Price5 | -0.333^***^  (0.095) |  |
| Price6 | -1.493^***^  (0.143) |  |
| *ASC* | -4.189^***^  (0.897) |  |
| *ASC**age1 |  |  |
| *ASC**age2 | -0.050  (0.620) |  |
| *ASC**age3 | -1.037  (0.815) |  |
| *ASC**age4 | -0.688  (0.883) |  |
| *ASC**gender | -0.261  (0.364) |  |
| *ASC**education1 |  |  |
| *ASC**education2 | -0.174  (0.111) |  |
| *ASC**education3 | -0.230  (0.539) |  |
| *ASC**average monthly income1 in 2019 |  |  |
| *ASC**average monthly income2 in 2019 | 0.201  (0.596) |  |
| *ASC**average monthly income3 in 2019 | 0.065  (0.660) |  |
| *ASC**average monthly income4 in 2019 | 0.885  (0.710) |  |
| *ASC**average monthly income5 in 2019 | -0.279  (0.760) |  |
| *ASC**work in a medical-related industry | -0.964  (0.724) |  |
| *ASC**marital status | 1.445^*^  (-0.850) |  |
| *ASC**children | -0.416  (0.817) |  |
| *ASC**region1 |  |  |
| *ASC**region2 | -0.462  (0.660) |  |
| *ASC**region3 | -0.915  (0.596) |  |
| *ASC**region4 | -1.242^*^  (0.736) |  |
| *ASC**region5 | -1.239  (0.877) |  |
| *ASC**region6 | 0.476  (0.795) |  |
| *ASC**residence | 0.079  (0.551) |  |
| *ASC**chronic disease | 0.435  (0.469) |  |
| Error component |  | -5.023^***^  (0.321) |
| LL | -4,072.684 |  |
| AIC | 8,219.4 |  |
| BIC | 8,469.1 |  |

Notes: (1) SE means standard errors; (2) SD means standard deviation; (3) ^*^p<0.1, ^**^p<0.05, ^***^p<0.01.

Appendix Table 13 The results of the RPL-EC model with interaction terms (price adopts effect coding)

| Attribute | RPL-EC model with effectiveness*adverse | | RPL-EC model with protection*adverse | |
| --- | --- | --- | --- | --- |
|  | Coefficient | SD | Coefficient | SD |
| Vaccine effectiveness | | | | |
| 65% (Controlled level) | | | | |
| 80% | 0.274^*^  (0.154) | 0.525^***^  (0.109) | 0.522^***^  (0.119) | 0.449^***^  (0.118) |
| 95% | 2.108^***^  (0.201) | 1.998^***^  (0.139) | 1.430^***^  (0.153) | 1.863^***^  (0.144) |
| Vaccine protection period | | | | |
| 1 year (Controlled level) | | | | |
| 2 years | 0.501^***^  (0.143) | 0.018  (0.970) | 1.288^***^  (0.160) | 0.014  (0.939) |
| 3 years | 0.073  (0.186) | 0.570^***^  (0.123) | -0.425^***^  (0.148) | 0.445^***^  (0.139) |
| Vaccine adverse reactions | | | | |
| Mild (Controlled level) | | | | |
| No | 0.988^***^  (0.103) | 0.342  (0.225) | 1.117^***^  (0.090) | 0.583^***^  (0.149) |
| Price1 (Controlled level) | | | | |
| Price2 | 0.154  (0.165) |  | 0.154  (0.231) |  |
| Price3 | 0.157  (0.128) |  | -0.436^**^  (0.187) |  |
| Price4 | 0.461^*^  (0.263) |  | 1.564^***^  (0.275) |  |
| Price5 | 0.044  (0.200) |  | -0.597^***^  (0.119) |  |
| Price6 | -1.537^***^  (0.164) |  | -1.623^***^  (0.157) |  |
| *ASC* | -4.776^***^  (0.348) |  | -5.162^***^  (0.357) |  |
| eff2*pro2 |  |  |  |  |
| eff3*pro2 |  |  |  |  |
| eff2*pro3 |  |  |  |  |
| eff3*pro3 |  |  |  |  |
| eff2*side2 | -0.542^**^  (0.238) |  |  |  |
| eff3*side2 | 0.419^**^  (0.207) |  |  |  |
| pro2*side2 |  |  | 0.802^***^  (0.158) |  |
| pro3*side2 |  |  | -0.273  (0.191) |  |
| Error component |  | -5.083^***^  (0.327) |  | -5.171^***^  (0.327) |
| LL | -4,081.776 |  | -4,069.125 |  |
| AIC | 8,201.6 |  | 8,176.2 |  |
| BIC | 8,329.8 |  | 8,304.5 |  |

Notes: (1) SD means standard deviation; (2) Standard errors (SE) in parentheses; (3) ^*^p<0.1, ^**^p<0.05, ^***^p<0.01.

Appendix Table 14 Class memberships from the ECLC-homogeneity model (price adopts effect coding)

| Class | Description ANA behavior | Probabilities |
| --- | --- | --- |
| Class 1 | AA (all attendance) | 31.82% |
| Class 2 | ANA-price (only price nonattendance) | 5.86% |
| Class 3 | ANA-adverse reactions (only adverse reactions nonattendance) | 5.16% |
| Class 4 | ANA-effectiveness + price (effectiveness and price nonattendance) | 12.38% |
| Class 5 | ANA-effectiveness + protection period + adverse reactions (only price attendance) | 14.41% |
| Class 6 | ANA-effectiveness + protection period + price  (only adverse reactions attendance) | 3.43% |
| Class 7 | ANA-protection period + adverse reactions + price (only effectiveness attendance) | 21.53% |
| Class 8 | ANA-effectiveness + adverse reactions + price  (only protection period attendance) | 5.41% |

Appendix Table 15 The results of the ECLC-homogeneity and ECLC-heterogeneity models (price adopts effect coding)

| Attribute | ECLC-homogeneity model | ECLC-heterogeneity model | | |
| --- | --- | --- | --- | --- |
|  | AA | AA1 | AA2 | |
|  | Coefficient | Coefficient | Coefficient | |
| Vaccine effectiveness | | | |  |
| 65% (Controlled level) | | | | |
| 80% | 0.581^***^  (0.103) | 0.638^***^  (0.133) | 2.132^***^  (0.433) | |
| 95% | 2.894^***^  (0.156) | 2.693^***^  (0.167) | 4.266^***^  (0.397) | |
| Vaccine protection period | | | |  |
| 1 year (Controlled level) | | | | |
| 2 years | 0.820^***^  (0.131) | 0.530^***^  (0.166) | 1.184^***^  (0.290) | |
| 3 years | 0.461^***^  (0.137) | 0.174  (0.157) | 0.173  (0.168) | |
| Vaccine adverse reactions | | | |  |
| Mild (Controlled level) | | | | |
| No | 1.576^***^  (0.139) | 0.989^***^  (0.126) | 0.843^***^  (0.153) | |
| Price1 (Controlled level) | | | | |
| Price2 | -0.217  (0.195) | -0.739^**^  (0.320) | 0.437  (0.267) | |
| Price3 | 0.153  (0.122) | 1.008^***^  (0.241) | 0.163  (0.348) | |
| Price4 | 0.825^***^  (0.156) | 1.354^***^  (0.305) | 0.912^**^  (0.380) | |
| Price5 | -0.166  (0.104) | -0.721^***^  (0.180) | 0.026  (0.452) | |
| Price6 | -1.509^***^  (0.164) | -2.112^***^  (0.322) | -2.144^***^  (0.511) | |
| *ASC* | -4.018^***^  (0.269) | -3.502^***^  (0.171) | 3.341^***^  (0.248) | |
| Class probability |  | 0.244^***^ | 0.084^***^ | |
| LL | -4,050.162 | -4,065.290 |  | |
| AIC | 8,150.3 | 8,186.6 |  | |
| BIC | 8,319.0 | 8,375.5 |  | |

Notes: (1) Standard errors (SE) in parentheses; (2) ^*^p<0.1, ^**^p<0.05, ^***^p<0.01.

Appendix Table 16 The probability of ANA (price adopts effect coding)

|  | ECLC-homogeneity model | ECLC-heterogeneity model |
| --- | --- | --- |
| Full attributes attendance | 31.82% | 32.76% |
| Effectiveness | 35.63% | 37.80% |
| Protection period | 39.37% | 21.10% |
| Adverse reactions | 46.51% | 23.40% |
| Price | 48.60% | 21.10% |
| ANA | 0.00% | 0.00% |

Appendix table 17 LL, AIC, and BIC information of the models (price adopts effect coding)

| Model | LL | AIC | BIC |
| --- | --- | --- | --- |
| RPL Model | -4,862.250 | 9,756.5 | 9,864.5 |
| RPL-EC Model | -4,083.806 | 8,201.6 | 8,316.3 |
| RPL-EC model (with sociodemographic characteristics) | -4,072.684 | 8,219.4 | 8,469.1 |
| RPL-EC model with effectiveness*adverse | -4,081.776 | 8,201.6 | 8,329.8 |
| RPL-EC model with protection*adverse | -4,069.125 | 8,176.2 | 8,304.5 |
| Latent class model (2 classes) | -4,508.384 | 9,062.8 | 9,218.0 |
| Nested logit model | -5,160.081 | 10,344.2 | 10,425.1 |
| ECLC-homogeneity model | -4,050.162 | 8,150.3 | 8,319.0 |
| ECLC-heterogeneity model | -4,065.290 | 8,186.6 | 8,375.5 |
