# Supplementary material for: Attribute nonattendance in COVID‐19 vaccine choice: A discrete choice experiment based on Chinese public preference
Source: Health Expect. 2022 Jan 20;25(3):959–70. doi: 10.1111/hex.13439 (PMC9122444; doi:10.1111/hex.13439)
Supplement: Supplementary file 2 — Supplementary information. [file HEX-25--s002.docx]

**COVID-19 vaccine attributes preference questionnaire**

## Questionnaire 1:

Dear interviewee:

Coronavirus disease 2019 (COVID-19) is an infectious disease caused by a newly discovered coronavirus—SARS-CoV-2 (World Health Organization). Globally, as of 4:35 p.m. on 27 Jan 2021, there have been 99,864,391 confirmed cases of COVID-19, including 2,149,700 deaths, and the pandemic is still spreading around the world (World Health Organization). To better understand the Chinese public’s attitudes toward the COVID-19 vaccine, we are conducting a survey of COVID-19 vaccine attribute preferences. This survey is anonymous. Your information and answers will be kept confidential, and all information will be used only for academic research. Thank you very much for your support!

### Section 1: Discrete choice experiment of the COVID-19 vaccine.

Assuming that a variety of Chinese COVID-19 vaccines have been developed and are available, you can be vaccinated at the nearest hospital. Each choice set below contains two types of vaccines. Please choose the vaccine that you prefer to receive according to your true wishes. If neither of the options is acceptable to you, then you can choose the “neither” option.

Please note that our hypothetical COVID-19 vaccine requires two injections, with an interval of 14 to 28 days between them. These vaccines differ in terms of effectiveness (65%/80%/95%), protection period (1 year/2 years/3 years), adverse reactions (mild adverse reactions/no adverse reactions) and price.

Note: Vaccine effectiveness refers to the percentage reduction in morbidity among vaccinated people compared to those who are not vaccinated. For example, if the morbidity is 10% in vaccinated people and 50% in unvaccinated people, then the vaccine effectiveness is (50%-10%)/50%=80%. The vaccine protection period refers to the length of time the vaccine induces individual immunity. Adverse reactions to the vaccine refer to reactions caused by the vaccine itself, which are irrelevant to the purpose of vaccination or unexpected and are related to individual differences. Mild adverse reactions manifest mainly as local pain, redness and swelling at the injection site, transient low-grade fever, fever, etc. Transient refers to a clinical symptom or sign that appears once in a short period of time, often with obvious incentives.

#### 1. Please choose the vaccine you prefer to get from the following two COVID-19 vaccines according to your true wishes.

|  | Vaccine A | Vaccine B | Neither |
| --- | --- | --- | --- |
| Effectiveness | 65% | 80% |  |
| Protection period | 1 year | 3 years |  |
| Adverse reactions | Mild adverse reactions | Mild adverse reactions |  |
| Price (Unit: ¥) | 50 | 300 |  |
| Which vaccine do you prefer? |  |  |  |

#### 2. Please choose the vaccine you prefer to get from the following two COVID-19 vaccines according to your true wishes.

|  | Vaccine A | Vaccine B | Neither |
| --- | --- | --- | --- |
| Effectiveness | 95% | 80% |  |
| Protection period | 1 year | 1 year |  |
| Adverse reactions | No adverse reactions | Mild adverse reactions |  |
| Price (Unit: ¥) | 300 | 200 |  |
| Which vaccine do you prefer? |  |  |  |

#### 3. Please choose the vaccine you prefer to get from the following two COVID-19 vaccines according to your true wishes.

|  | Vaccine A | Vaccine B | Neither |
| --- | --- | --- | --- |
| Effectiveness | 80% | 95% |  |
| Protection period | 2 years | 2 years |  |
| Adverse reactions | Mild adverse reactions | No adverse reactions |  |
| Price (Unit: ¥) | 50 | 200 |  |
| Which vaccine do you prefer? |  |  |  |

#### 4. Please choose the vaccine you prefer to get from the following two COVID-19 vaccines according to your true wishes.

|  | Vaccine A | Vaccine B | Neither |
| --- | --- | --- | --- |
| Effectiveness | 65% | 80% |  |
| Protection period | 2 years | 1 year |  |
| Adverse reactions | Mild adverse reactions | Mild adverse reactions |  |
| Price (Unit: ¥) | 500 | 1,000 |  |
| Which vaccine do you prefer? |  |  |  |

### Section 2: Sociodemographic information.

#### 5. Year of birth: ______.

#### 6. Gender:

1. Male;
2. Female.

#### 7. What is your educational background?

1. Primary school;
2. Junior high school and below;
3. Senior high school/Technical secondary school/Technical school;
4. Junior college;
5. Undergraduate college;
6. Master degree or above.

#### 8. Your monthly income in 2019?

1. ≤¥2,000;
2. ¥2,001-¥3,000;
3. ¥3,001-¥4,000;
4. ¥4,001-¥5,000;
5. ¥5,001-¥6,000;
6. ¥6,001-¥7,000;
7. ¥7,001-¥8,000;
8. ¥8,001-¥9,000;
9. ¥9,001-¥10,000;
10. ¥10,001-¥12,000;
11. ¥12,001-¥14,000;
12. ¥14,001-¥16,000;
13. ¥16,001-¥18,000;
14. ¥18,001-¥20,000;
15. ≥¥20,001.

#### 9. Your occupation (or work experience)

1. Medical-related industries;
2. Others.

#### 10. Your marital status:

1. Unmarried (Please jump to question 13);
2. Married;
3. Divorced/Widow.

#### 11. How many children do you have?

1. 0;
2. 1 child;
3. 2 children or more.

#### 12. Your residence is ______.

#### 13. Where are you staying currently?

1. Urban;
2. Rural.

#### 14. Do you have the following chronic diseases? [You can choose more than one answer.]

1. No chronic diseases;
2. Asthma;
3. Gout;
4. Cancer;
5. Diabetes;
6. Hypertension;
7. Coronary heart disease;
8. Cerebral infarction;
9. Stenocardia;
10. Hypothyroidism or hyperthyroidism;
11. Other: _________.

## Questionnaire 2:

Questionnaire 2 is similar to questionnaire 1 except for the four questions in Section 1.

#### 1. Please choose the vaccine you prefer to get from the following two COVID-19 vaccines according to your true wishes.

|  | Vaccine A | Vaccine B | Neither |
| --- | --- | --- | --- |
| Effectiveness | 80% | 95% |  |
| Protection period | 3 years | 2 years |  |
| Adverse reactions | No adverse reactions | Mild adverse reactions |  |
| Price (Unit: ¥) | 500 | 1,000 |  |
| Which vaccine do you prefer? |  |  |  |

#### 2. Please choose the vaccine you prefer to get from the following two COVID-19 vaccines according to your true wishes.

|  | Vaccine A | Vaccine B | Neither |
| --- | --- | --- | --- |
| Effectiveness | 65% | 95% |  |
| Protection period | 3 years | 1 year |  |
| Adverse reactions | No adverse reactions | Mild adverse reactions |  |
| Price (Unit: ¥) | 200 | 500 |  |
| Which vaccine do you prefer? |  |  |  |

#### 3. Please choose the vaccine you prefer to get from the following two COVID-19 vaccines according to your true wishes.

|  | Vaccine A | Vaccine B | Neither |
| --- | --- | --- | --- |
| Effectiveness | 80% | 65% |  |
| Protection period | 2 years | 1 year |  |
| Adverse reactions | Mild adverse reactions | No adverse reactions |  |
| Price (Unit: ¥) | 100 | 100 |  |
| Which vaccine do you prefer? |  |  |  |

#### 4. Please choose the vaccine you prefer to get from the following two COVID-19 vaccines according to your true wishes.

|  | Vaccine A | Vaccine B | Neither |
| --- | --- | --- | --- |
| Effectiveness | 65% | 95% |  |
| Protection period | 3 years | 3 years |  |
| Adverse reactions | No adverse reactions | Mild adverse reactions |  |
| Price (Unit: ¥) | 1,000 | 100 |  |
| Which vaccine do you prefer? |  |  |  |
